# Pubertal Health Group Counseling Based on Problem‐Solving, for Body Image Concerns and Anxiety in Adolescent Girls

**DOI:** 10.1002/brb3.70639

**Published:** 2025-07-03

**Authors:** Sana Nazmi, Hossein‐Ali Nikbakht, Fereshteh Behmanesh, Zeinab Gholamnia Shirvani, Alireza Azizi

**Affiliations:** ^1^ Student Research Committee Babol University of Medical Sciences Babol Iran; ^2^ Social Determinants of Health Research Center, Health Research Institute Babol University of Medical Sciences Babol Iran; ^3^ Clinical Research Development Unit, Shahid Yahyanezhad Hospital Babol University of Medical Sciences Babol Iran

**Keywords:** adolescents, anxiety, body image, puberty

## Abstract

**Background:**

Body image concerns often arise during puberty. The aim of this study is to determine the effectiveness of pubertal health group counseling based on problem‐solving for body image concerns and anxiety in adolescent girls.

**Methods:**

This quasi‐experimental study was conducted on 100 adolescent female students in Babol City during 2022–2023. The intervention group participated in weekly 90‐min problem‐solving group counseling on puberty health for 6 weeks. Data collection utilized socio‐demographic questionnaires, Littleton's Body Image Concern Inventory, and the Zung Anxiety Scale.

**Results:**

The mean difference in reduced body image concerns and anxiety before, immediately after, and 6 weeks post‐study in the problem‐solving consulting group was statistically significant (*p *< 0.005), unlike the control group. In the intervention group, the mean differences in body image concern were −21.40 (95% CI = −17.91 to −24.89) before to immediately, and −18.76 (95% CI = −15.07 to −22.44) before to 6 weeks later (*p* < 0.001). The average anxiety differences for these intervals were −9.68 (95% CI  = −6.83 to −12.52) and −8.52 (95% CI  = −6.06 to −19.97) in the intervention group (*p* < 0.001). The ANCOVA test results indicated a difference in body image concern and anxiety scores between the two groups after the study, accounting for baseline values with a reduction observed in the counseling group (*p* < 0.001). The effect sizes of body image concern and anxiety were 0.56 and 0.41, respectively.

**Conclusions:**

The findings demonstrate that problem‐solving group counseling effectively reduces body image concerns and anxiety in adolescent girls. Policymakers can leverage this research for health planning in counseling for this demographic.

**Trial Registration:**

Iranian Registry of Clinical Trials: (IRCT20180218038783N3)

## Graphical abstract

This quasi‐experimental study evaluated the effectiveness of problem‐solving group counseling on body image concerns and anxiety among adolescent girls. Participants receiving routine education plus problem‐solving counseling showed significant improvements compared to the control group, as measured by Littleton's Body Image Concern Inventory and the Zung Anxiety Scale. Problem‐solving group counseling combined with routine education significantly reduced body image concerns and anxiety levels among adolescent girls in a quasi‐experimental study, indicating its potential as an effective intervention for pubertal mental health.

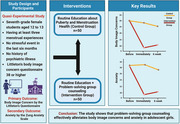

## 1 Introduction

The term “adolescence” is derived from the Latin word adolēscere, meaning to grow up (Santamaria 2020), which is associated with growth and change in biological, cognitive, social, and emotional aspects (Alsadat Fatemi et al. 2022; Nazarpour et al. 2020). During this period, adolescents achieve their true identity (Keshavarze 2020). In fact, many physical, psychological, and social problems and unhealthy behaviors have their roots in adolescence (F. Mastorci et al. 2024).

Currently, over 50% of the global population is under 25 years old, with one‐fifth (500 million) being adolescents (Ziapour et al. 2020). According to the 2015 census in Iran, approximately 7% of the population are teenagers aged 10–14, with 48% being girls (Z. Kazemi et al. 2020).

Paying attention to the health of adolescents is a crucial matter related to public health, and the World Health Organization has made the educational needs of adolescent girls a priority (George et al. 2018). A study by the Iranian Ministry of Health found that more than half of adolescents aged 10–14 lack awareness or have incomplete knowledge about puberty signs (Golchin et al. 2012; Mohammadi et al. 2014). A study in Iran revealed insufficient knowledge among adolescents, families, and educators about puberty and adolescent characteristics (Darabi et al. 2017). Health education is the third crucial need for adolescents (Panjalipour et al. 2020).

If adolescents have the required knowledge about the physiological changes of puberty, they might be experience a safe transition from adolescence to adulthood (Nazarpour et al. 2020; Nouri 2010). But regrettably, because of shame, taboos, and societal cultural beliefs, adequate education within the family setting, schools, and the incorporation of such topics in textbooks have been overlooked (Panjalipour et al. 2020). In numerous developing nations, numerous teenagers stay silent due to cultural influences and a lack of proper knowledge about pubertal health (Haque et al. 2014).

The body image concern or negative body image is one of the problems that may appear in a person at the same time as puberty (Bassaknezhad and Ataie Moghanloo 2013). Body image concern is acknowledged as a pervasive problem experienced by a large proportion of society (Sharifi et al. 2016). In a review study, it was found that 30%–75% of Iranians have body image concerns (Shoraka et al. 2019). Body image concern reaches their maximum during puberty, especially between the ages of 12 and 19 (Barkhordari‐Sharifabad et al. 2020; Ataie Moghanloo et al. 2014). A total of 88% of teenage girls have negative feelings about their body shape and size (Salarian Kaleji et al. 2022). In a study of 396 adolescent girls in Iran, 106 participants (26.8%) had body image concerns. A total of 89 individuals (84%) experienced moderate concerns, while 17 individuals (16%) had severe body image issues. These findings indicate that over one‐fifth of the adolescent girls studied had significant body image concerns (Baharvand et al. 2020). Adolescent girls' concern about their body image, whether based on reality or imagination, can lead to feelings of shame, resulting in avoidance of social interactions and academic difficulties. The physical, psychological, and social impacts of body image concerns, as well as the prevalence of this issue in Iran, are significant (Shoraka et al. 2019).

Structural changes of puberty are considered a crisis that may induce and continue adolescent problems such as increased anxiety. Body image concern evaluation is the predicting variable of anxiety (Raghibi et al. 2019). Due to an individual's distorted perception, emotions, and beliefs about their body, it can result in anguish, depression, and anxiety disorders (Junne et al. 2016), as well as a decrease in quality of life (Crerand et al. 2017), weight concerns (Bibiloni et al. 2017) and eating disorders (Kristjánsdóttir et al. 2019).

Anxiety disorders are the most common mental health disorders that affect adolescents (Bassaknezhad and Ataie Moghanloo 2013; Reardon et al. 2009). The World Health Organization reports that 3.6% of 10–14‐year‐olds and 4.6% of 15–19‐year‐olds suffer from an anxiety disorder (WHO 2021). In a study in Iran, 61.7% of female students experienced medium and severe degrees of anxiety during their puberty period (Mokari et al. 2016). Recognizing the importance of education and puberty health can prevent many problems for adolescents and reduce their distress (Mokari et al. 2016). It is very important to hold health education appropriate for adolescents needs using new educational methods and reliable resources (Kheyrkhah et al. 2013).

Regarding puberty health counseling, various pieces of training have been used, including training based on planned behavior (Eslamimehr et al. 2017), health belief model (Kheirollahi et al. 2017), and lecture‐based instructional approach (Nazarpour et al. 2020), but there have been contradictory results in different studies. In the lecture method, the development of cognitive traits may progress slowly and not adequately enhance advanced knowledge, particularly in practical skills and behavioral changes (Kay et al. 2019).

Counseling has a great impact on the health of adolescent girls and their social role in the future (Ziapour et al. 2021; Nozari et al. 2019). Group counseling is one of the available methods to address the problems and concerns of adolescents. In group counseling, which is a two‐way process, the counselor examines problems, feelings, attitudes, and values (Hoseini et al. 2021). This process will help them to have a correct perception of themselves and their surrounding world (Khazaie and Riahi 2021).

One of the puberty counseling methods is problem‐solving skills, which has a fundamental role in reducing interpersonal and intrapersonal conflicts and problems (Koumleh et al. 2017). Those who do not have problem‐solving skills may feel frustrated when faced with problems (Rahbari et al. 2017). Problem‐solving includes: defining the problem, presenting existing solutions, making decisions, implementing solutions, and finally evaluating the results (Hoseini et al. 2021; Michelson et al. 2020). Therefore, it can be a more effective method for puberty health counseling. Hoseini et al. (2021) conducted a study on the impact of group counseling using problem‐solving techniques on pubertal health in adolescent girls, revealing a positive effect of this approach on pubertal health.

In Iran, most adolescents, especially girls, are deprived of accurate information about puberty changes due to cultural issues (Mazloomy Mahmoodabad et al. 2013) and they typically only receive 1 h of general education on puberty health. This limited time may not be sufficient for their needs. Most of the studies on adolescent health and puberty in girls have been descriptive and there have not been many experimental studies on the effect of counseling (Nozari et al. 2019). The present study aims to determine the effectiveness of pubertal health group counseling based on problem‐solving on body image concerns and anxiety in adolescent girls in Babol. Although, anxiety was investigated as a secondary variable.

## 2 Methods

This quasi‐experimental study was approved by the ethics committee of Babol University of Medical Sciences (IR.MUBABOL.HRI.REC.1401.222), and the Iranian Registry of Clinical Trials (IRCT20180218038783N3) on 24/02/2023 (Available at: https://www.irct.ir/search/result?query. https://irct.behdasht.gov.ir/user/trial/68598/view. The correct link = IRCT20180218038783N3). The study protocol of this article publish in the *Journal of Education and Health Promotion* (Nazmi et al. 2024).

The research was conducted in girls' secondary schools in Babol City, involving seventh‐grade female students aged 12–13 (Djalalinia et al. 2012). The sample size was determined according to the research objectives, and the previous study (Barkhordari‐Sharifabad et al. 2020), considering the comparison of the mean and SDs, the confidence level of 95%, the power of 95, and the effect size of 1.08 using G‐Power software Version 3. At least 14 samples were required in each group, but 45 samples in each group were considered to perform the final ANCOVA analysis and to examine the outcome measures with regard to the baseline value and the influencing factor. Considering the 10% drop, 100 samples were determined as the final sample size (50 samples in each group).

n=Z1−αα22+Z1−β2S2μ1−μ22

Multistage sampling was used to obtain the unit of analysis. In this study, at least four secondary schools in Babol City were selected. In terms of municipal divisions, the city of Babol has two districts, each one being considered a social class. In the first stage, two schools were randomly selected from each district. Next, one school was assigned to the control group and one to the intervention group in each district. To reduce potential information exchange between students, study groups were randomly chosen from different schools. This study employed randomization without random allocation due to the sampling method.

Inclusion criteria include the following: students' willingness to participate in the study, parents' consent for their children's participation, approval of students’ participation by the school, living with parents, having at least three menstrual experiences, no stressful event in the last 6 months (such as the death of a first‐degree family member), no history of psychiatric illness or taking psychiatric drugs, no skeletal diseases and obtaining a score of 38 or higher on Littleton's body Image Concern Questionnaire. The focus on 12–13‐year‐old girls aligns with the initiation of puberty health education programs in Iran, targeting a key developmental period for addressing body image and anxiety concerns. While acknowledging potential developmental differences across adolescence, this age range provided a homogenous sample for a controlled study of our intervention. Also, specifically included girls with at least three menstrual cycles to ensure that all participants had established menstruation. This was crucial because several questions in the assessment tools related to menstrual hygiene practices and dietary considerations during menstruation. Therefore, it was essential to confirm that participants had direct experience with menstruation to provide informed and accurate responses to these questions. Exclusion criteria include: not responding to 10% of the questions in the questionnaire and unwillingness to continue participating in the study (Nulty 2008).

The researcher went to each school, introduced himself to the school officials, and explained the objectives of the research. Among the 253 students, 23 were excluded due to a lack of inclusion criteria. A total of 230 students completed the demographic inventory, Littleton's Standard Body Image Concern Questionnaire, and Zung's anxiety questionnaires. A total of 71 students were excluded from the study because they scored less than 38 on the Littleton Body Image Concern Questionnaire. The final samples were selected from students who scored 38 and above (low to severe anxiety) from the standard body image concern questionnaire, using random sampling (Flowchart 1).

Before completing the questionnaires by students, their parents’ consent was obtained. For the intervention group, the researcher held an orientation session for the students who were willing to participate and had the inclusion criteria. Before the implementation of the study, the educational content for adolescent health based on problem‐solving was prepared. This content was designed according to the educational content of puberty health in different dimensions, including the age of earliest signs of puberty, reproductive system, physical changes of puberty, menstrual cycle and health, dysmenorrhea, nutrition during puberty, and psychological symptoms of puberty (such as premenstrual syndrome (PMS), methods of dealing with anxiety and reducing body image concern, identity formation, affective and emotional issues, psychological disorders, signs of personality development, socio‐moral development, intellectual and mental development in different periods of time). It was also prepared based on the educational package of the Ministry of Health, Treatment and Medical Education and reviewing available resources, using the advice of a psychologist for problem‐solving skills. Puberty health training was in three domains of cognitive, emotional, and behavioral. Then, seven experts evaluated its content validity.

Then, six sessions of counseling and group training based on problem‐solving were implemented for the intervention group of each school separately. These sessions were done by the researcher (counseling in midwifery student) and using a computer, video projector, PowerPoint presentations, lectures, and questions and answers. Training sessions were held once a week for 90 min. In the intervals between face‐to‐face meetings, the researcher was in touch with the students of the intervention group through social networks (Eitaa) and answered their questions about puberty health. To ensure consistency across all schools, a single, trained counselor conducted all counseling sessions. This approach minimized variability in the intervention's delivery. The problem‐solving steps are as follows:

1. Present the problem and encourage students to clarify unclear points.
2. Explain the topic to the participants.
3. Conduct brainstorming, group participation, and discussions about the topic.
4. List facts, make hypotheses based on the problem content, and answer questions to better achieve educational goals.
5. Reach intragroup agreement on learning goals and ensure the facilitator's commitment to achieving complete, comprehensive, and appropriate goals (determination of learning topics).
6. Engage students in individual and group study to gather information from introductory sources.
7. Present topics based on hypotheses, goals, and questions, and facilitate group discussions.
8. Summarize and evaluate the presented topics as the facilitator (Table 1).

**TABLE 1:** Intervention sessions.

Session number (time)	The problem presented	Problem‐solving steps
First session (90‐min)	‐Guidelines for conducting problem‐solving meetings ‐Maturation in general ‐Onset age of puberty signs ‐Reproductive system ‐Physical indicators of puberty	1. Problem presentation (generalities and onset age of puberty signs) 2. Problem description 3. Flow of thoughts 4. Hypothesis formation 5. Subject of study determination
The time gap between the initial and subsequent sessions		Individual and group study (generalities and age of onset of puberty signs and reproductive system, physical signs of puberty)
Second session (90‐min)	‐Menstruation and its hygiene ‐ Dysmenorrhea	7. Sharing content (generalities and age of onset of puberty signs and reproductive system, physical signs of puberty) 8. Problem‐solving, conclusion, and summary 1. Presentation of the issue (menstruation and hygiene, dysmenorrhea) 2. Problem description 3. Stream of consciousness 4. Formulating hypotheses 5. Identifying the learning topic
The time interval between the second and third session		6. Individual and group study (menstruation and its hygiene, dysmenorrhea)
Third session (90‐min)	‐Psychological symptoms of puberty (PMS) ‐Nutrition during puberty	7. Sharing information (menstruation and its hygiene, dysmenorrhea) 8. Problem‐solving, conclusion and summary 1. Introducing the issue (psychological symptoms of puberty (PMS), nutrition during puberty) 2. Description of the problem 3. Rain of thoughts 4. Formulating hypotheses 5. Determination of learning topics
The time interval between the third and fourth session		6. Individual and group study (psychological symptoms of puberty (PMS), nutrition during puberty)
Fourth Session (90‐min)	‐Psychological signs of puberty (anxiety and concern about body image) 1. 2. Explaining the problem 3. Delving into various thoughts 4. Formulating hypotheses 5. Identifying the focus of study	7. Addressing the psychological symptoms of puberty (PMS) and nutritional needs during this stage 8. Problem‐solving, conclusion and summary 1. Introducing the issue of psychological symptoms during puberty, such as anxiety and body image concerns 2. Explaining the problem 3. Rain of thoughts 4. Formulating hypotheses 5. Identifying the focus of study
The time interval between the fourth and fifth sessions		6. Individual and group study of psychological symptoms of puberty (anxiety and concern about body image)
Fifth Session (90‐min)	‐Psychological signs of maturity (identification, emotional and emotional issues, mental disorders, signs of personality development, social, moral and intellectual and mental development in different periods of time) Psychological signs of puberty include identification, emotional and mental disorders, personality development, and social, moral, and intellectual growth across various life stages. ‐Local customs and beliefs, along with the Islamic religious guidelines concerning the puberty of girls	7. Sharing content (psychological signs of puberty, anxiety and concern about body image) 8. Problem‐solving, conclusion and summary 1. Problem presentation (psychological signs of puberty, local customs and beliefs, Islamic religious rules on girls' puberty) 2. Problem description 3 Rain of thoughts 4. Formulating hypotheses 5. Identifying the learning subject
The time interval between the fifth and sixth sessions		6. Individual and group study (local customs and beliefs, orders of the Islamic religion regarding the puberty of girls), psychological signs of puberty (identification, emotional and emotional issues, mental disorders, signs of personality development, social, moral and intellectual and mental development in the courses different time)
The sixth session (90‐min)	‐Review past sessions	7. Exploring local customs and beliefs, Islamic directives on girls' puberty, psychological markers of puberty (recognition, emotional and cognitive challenges, mental health aspects, indications of character growth, social, moral, intellectual, and psychological evolution through various stages). 8. Problem‐solving, conclusion and summation

Meanwhile, the students in the control group did not receive any educational intervention from the researcher and they remained on a waiting list. Both the intervention and control groups received the routine education about puberty and menstruation health for about an hour from the trained midwives of the health center. This training is provided in one session for all students in Iran based on the instructions of the Department of School Health at the Health Care Deputy each year.

At the end of the intervention, the educational package provided to the intervention group was given to the students of the control group to maintain the ethical considerations.

The primary outcome was body image concern and the secondary outcome was anxiety. Both the intervention and control groups completed Littleton's Body Image Concern and Zung's anxiety questionnaires immediately after the study and 6 weeks later. In this study, to minimize social desirability, the researcher conducting the training and counseling was different from the one distributing the questionnaires to the students.

The data collection method consisted of three parts. The first part was a personal and social information questionnaire including age, parents' age, parents' education, parents' occupation, place of residence, number of family members, birth order, and previous information about puberty, the source of puberty information, and the preferred source of information. The second part of the questionnaire consisted of Littleton's Body Image Concern Inventory (BICI), which includes 19 five‐point Likert scale items ranging from 1 (*never*) to 5 (*always*). This instrument is designed to assess cognitive, emotional, and behavioral concerns related to body image, particularly focusing on dissatisfaction with appearance, preoccupation with perceived flaws, and compulsive appearance‐checking behaviors. Example items include: “I often check my appearance in a mirror” and “I worry a lot about how my body looks to others.” The internal consistency of this scale in the present study was high, with a Cronbach's alpha of 0.93. Item‐total correlations ranged from 0.32 to 0.72, with an average of 0.62 (Littleton et al. 2005; Basak Nejad and Ghaffari 2007).

The third part comprised Zung's Self‐Rating Anxiety Scale (SAS), which contains 20 items measured on a 4‐point Likert scale ranging from 1 (*never*) to 4 (*almost always*), with selected items reverse‐scored (Items 5, 9, 13, and 19). This scale measures a range of anxiety‐related symptoms, including physiological, affective, and cognitive dimensions of anxiety. Representative items include: “I feel more nervous and anxious than usual” and “I have trouble sleeping at night.” The scale demonstrated good reliability in this study, with a Cronbach's alpha of 0.81. Item‐total correlations ranged from 0.34 to 0.65 (Sahebi and Salari 2005; Zung 1971; Olatunji et al. 2006).

For statistical analysis, students' descriptive information was reported using the mean and standard deviation or in the form of numbers and percentages. To check the statistical tests, the Kolmogorov–Smirnov test was first used to examine the normality of the data. Then if it was normal, parametric tests were used. The Chi‐square test was used to examine the relationship between two qualitative variables. The conditions for the Chi‐square test were not met, Fisher's test was used.

Independent Samples *t*‐test was used to compare the equality of two sample means between qualitative variables with the assumption of equality of variances. The repeated measures ANOVA was used to compare the means across quantitative variables in more than two dependent situations. Also, the ANCOVA test was considered to compare the changes in students' concerns and anxiety scores after the intervention in the two groups, taking into account the pre‐intervention and potential confounding variables measurements. The standardized mean difference (SMD) with a 95% confidence interval was reported for effect size. In the study, the SMD index according to Cohen's *d* was used (Lenhard and Lenhard 2022). All analyses were performed using SPSS software version 25 and the significant level was considered (*p* < 0.05) for all analyses.

## 3 Results

In this study, 100 qualified students were included and divided into two intervention and control groups (50 samples in each group). Table 2 indicates the results of the demographic characteristics of the research units reported in terms of groups. The results showed that the two studied groups had no statistically significant difference in terms of variables such as age, parents' education, father's job, place of residence, birth order, number of family members, source of information, preferred source of information, and parent's age. But there was a difference between the two groups in terms of the mother's job and previous puberty information variables.

**TABLE 2:** Demographic characteristics of the research units divided by groups (before the intervention).

Variable name	Variable subgroup	Total(number)	Groups		*p* value
			Intervention number (%)	Control number (%)	
**Age** ^a^	12	49	26 (53.1)	23 (46.9)	0.548
	13	51	24 (47.1)	27 (52.9)	
**Father's education** ^a^	Diploma and less	78	38 (48.7)	40 (51.3)	0.629
	University	22	12 (54.5)	10 (45.5)	
**Mother's education** ^a^	Diploma and less	83	39 (47.0)	44 (53.0)	0.183
	University	17	11 (64.7)	6 (35.3)	
**Father's job** ^b^	Freelance job	71	36 (50.7)	35 (49.3)	0.240
	Manual worker	9	2 (22.2)	7 (77.8)	
	Employee	10	7 (70.0)	3 (30.0)	
	Other	10	5 (50.0)	5 (50.0)	
**Mother's job** ^a^	Housewife	70	28 (40.0)	42 (60.0)	0.002
	Employed	30	22 (73.3)	8 (26.7)	
**Place of residence** ^b^	Village	9	5 (55.6)	4 (44.4)	1.000
	City	91	45 (49.5)	46 (50.5)	
**Previous puberty information** ^a^	No	10	2 (20.0)	8 (80.0)	0.046
	Yes	90	48 (53.3)	42 (46.7)	
**Source of information** ^b^	No answer	5	2 (40.0)	3 (60.0)	0.401
	Mother	55	31 (65.4)	24 (43.6)	
	Non‐mother	40	17 (42.5)	23 (57.5)	
**Preferred source of information** ^a^	Mother	64	31 (48.4)	33 (51.6)	0.677
	Non‐mother	36	19 (52.8)	17 (47.2)	
**Parents' age** ^c^ **(year)**	Father	100	**Mean ± standard deviation**	**Mean ± standard deviation**	0.665
			44.02 ± 5.31	43.54 ± 5.72	
	Mother	100	37.76 ± 4.92	38.68 ± 5.15	0.364

^a^ Chi‐square.

^b^ Fisher's exact test.

^c^ Independent samples *t‐*test

To analyze the data, we employed two strategies:

1. In the initial approach, we compared the mean difference of body image concern and anxiety across three distinct time periods in both intervention and control groups. The mean difference, confidence interval, and *p* value were documented for each group.
2. The second strategy involved evaluating the difference between the averages of each intervention and control group, and comparing the results between the two groups.

**FIGURE 1:**
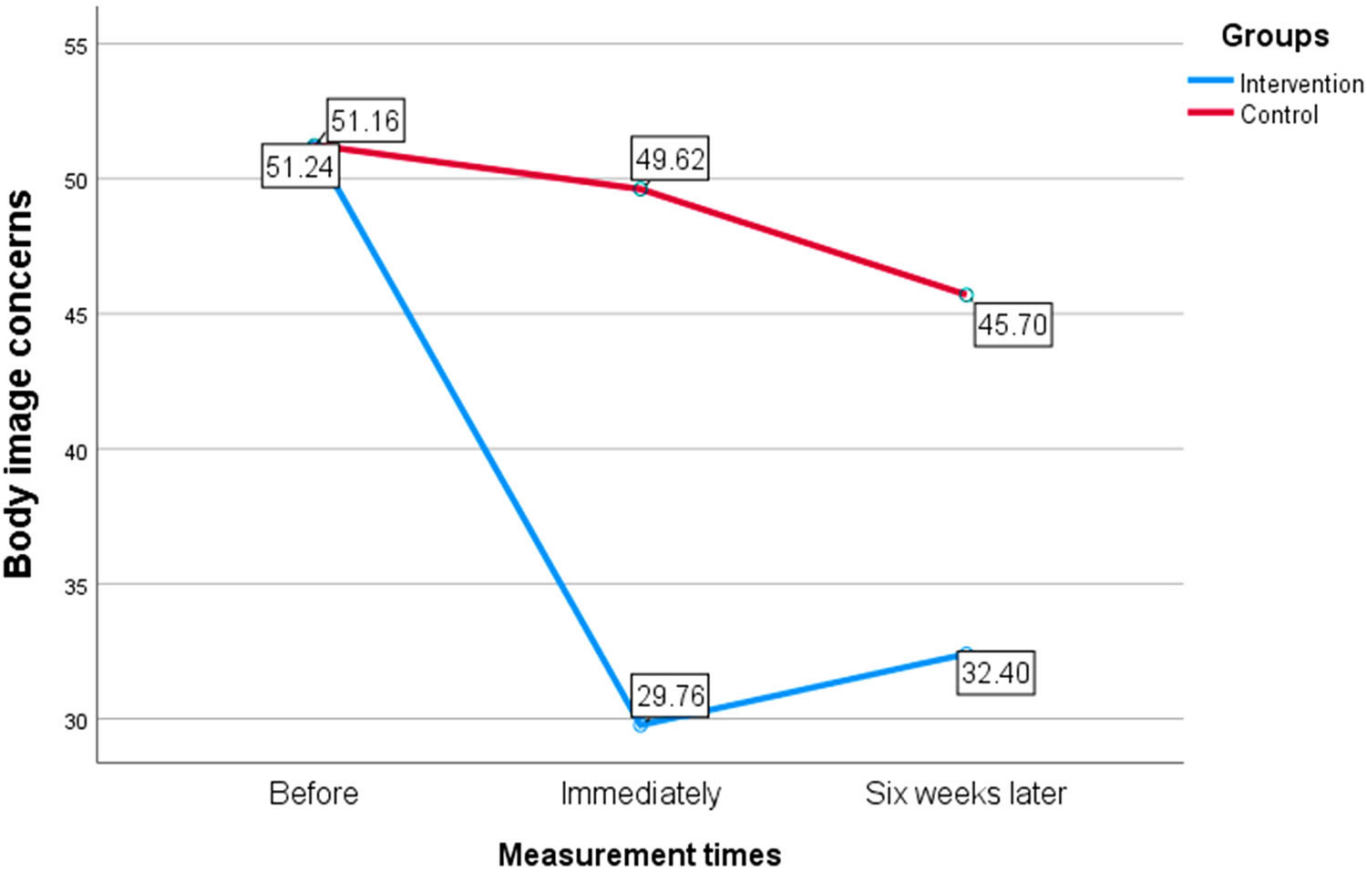
The average body image concern score in two intervention and control groups in the three time periods examined in the study.

In the initial strategy, within the problem‐solving counseling intervention group, a decrease in concern regarding body image was noted. Comparing the period before and immediately after the intervention showed a reduction of −21.40 units, while comparing the time before and 6 weeks after the intervention showed a reduction of about −18.76 units. It was statistically significant (*p* < 0.001). According to the values mentioned in SMD, the reduction of body image concerns in students of the intervention group is clinically significant (Lenhard and Lenhard 2022). However, in the control group, when comparing various time periods, the mean difference obtained was minimal, although comparing the time before and 6 weeks after the intervention, the *p* value was significant, but compared to the changes in the intervention group, it was very small, and compared to the two in another time period (before and 6 weeks later and immediately and 6 weeks later) the obtained value was not statistically significant (Table 3). Figure 1 depicts the average body image concern scores in two intervention and control groups across the three time periods analyzed. It illustrates a notable reduction in body image concern within the intervention group in comparison to the control group.

**TABLE 3:** Examining the mean difference in body image concern and anxiety in three different time periods in intervention and control groups.

Variable name	Groups	Time group	Mean difference	Confidence interval (95% CI)	Standard mean difference (95% CI)	*p* value pairwise comparisons	*p* value total
**Body image concerns**	Intervention	Before‐immediately	−21.40	−24.89 to −17.91	−2.51 (−3.14 to −2.09)	< 0.001	< 0.001
		Before—6 weeks later	−18.76	−22.44 to −15.07	−2.20 (−2.75 to −1.76)	< 0.001	
		Immediately—6 weeks later	2.64	−0.22 to 5.30	0.33 (−0.06 to 0.72)	0.053	
	Control	Before‐immediately	−1.62	−4.66 to 1.42	−0.15 (−0.53 to 0.24)	0.478	0.003
		Before—6 weeks later	−5.54	−9.91 to −1.16	−0.45 (−0.84 to −0.04)	0.009	
		Immediately—6 weeks later	−3.92	−7.75 to −0.08	−0.34 (−0.70 to 0.08)	0.044	
**Anxiety**	Intervention	Before‐immediately	−9.68	−12.52 to −6.83	−1.03 (−1.60 to −0.77)	< 0.001	< 0.001
		Before—6 weeks later	−8.52	−10.97 to −6.06	−0.91 (−1.40 to −0.58)	< 0.001	
		Immediately—6 weeks later	1.16	−0.74 to 3.06	−0.21 (−0.21 to 0.56)	0.359	
	Control	Before‐immediately	1.16	−1.70 to 4.02	0.11 (−0.27 to 0.51)	0.687	0.299
		Before—6 weeks later	−0.66	−3.49 to 2.17	−0.06 (−0.46 to 0.32)	0.919	
		Immediately—6 weeks later	−1.82	−4.85 to 1.21	−0.21 (−0.60 to 0.18)	0.374	

*Note*: Repeated measures ANOVA and Tukey's post hoc test for pairwise comparisons.

**FIGURE 2:**
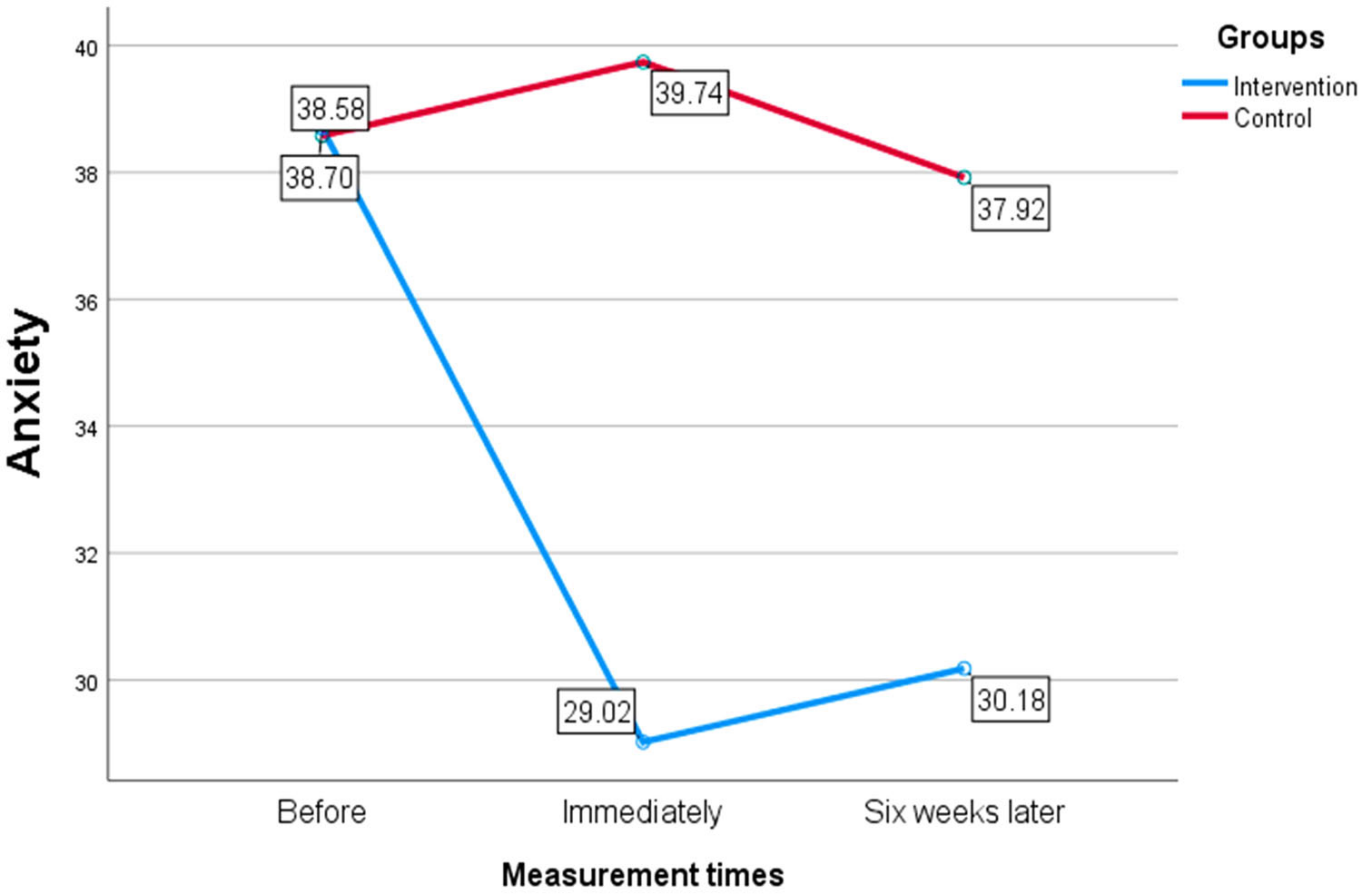
The average anxiety score in two intervention and control groups in the three time periods examined in the study.

In the intervention group, anxiety levels decreased by approximately 10 units immediately after the intervention and by about 9 units 6 weeks later, compared to baseline. It was statistically and clinically significant and is very valuable in order to reduce the level of anxiety in adolescents. In comparing the time immediately after the intervention and 6 weeks later, no significant findings were obtained. In the control group, anxiety levels did not significantly decrease in any of the two time periods. This analysis focused on examining and documenting the mean difference within each group (Table 3). Figure 2 displays the average anxiety scores of two intervention and control groups across the three time periods examined in the study. A notable decrease in anxiety levels is evident in the intervention group compared to the control group.

**FLOWCHART 1:**
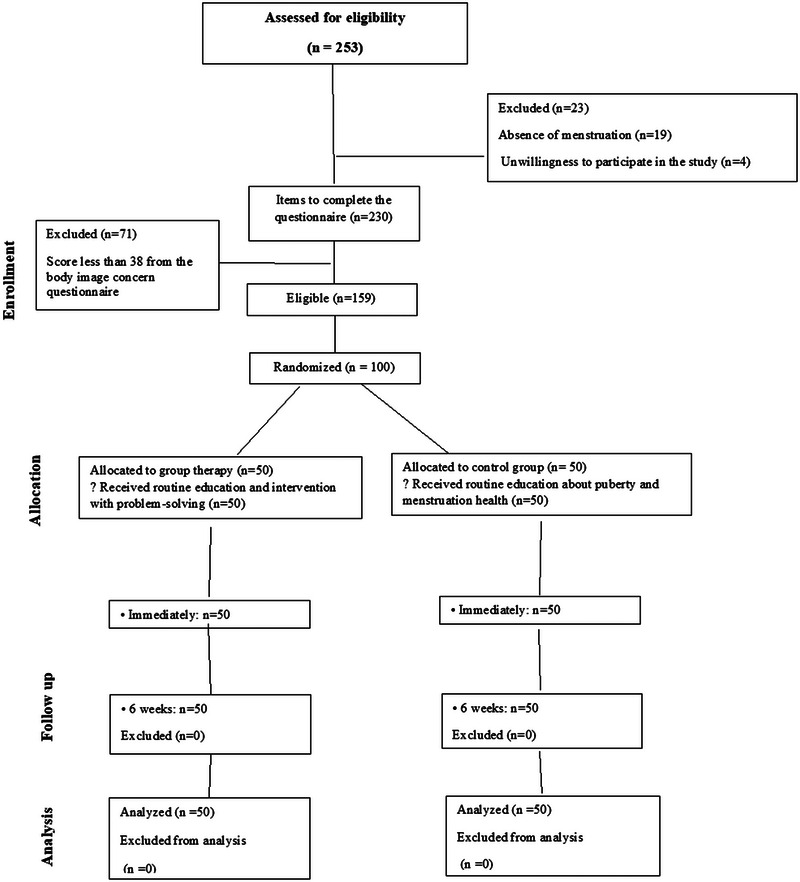
Flow of participants through the study.

In the second analysis strategy, after calculating the mean difference in each group, the mean difference obtained in Table 3 was compared between the intervention and control groups. The findings indicated that the mean difference between before and after the study in both groups for the body image concern variable, before and immediately, before and 6 weeks after the study decreased by about 20 and 13 units, in the intervention group respectively, which was statistically significant (*p* < 0.001). Immediately and 6 weeks after the study, mean difference was 6.56, which was statistically significant (*p* < 0.001). Regarding the variable of anxiety, the mean difference between the two groups before and immediately after the study was about 11 units decreased in intervention group, before and 6 weeks after the study, about 8 units decreased in the intervention group, which was statistically significant (*p* < 0.001). Also, the mean difference between the two groups immediately and 6 weeks after the study was 2.98, which was not statistically significant (*p* = 0.043) (Table 4).

**TABLE 4:** Comparison of the mean difference between before and after study in both groups for the variables of body image concern and anxiety in three time periods (absolute effect size of the study in the measured times).

Variable name	Time group	Mean difference	Confidence interval(95% CI)	Effect size(95% CI)	*p* value
**Body image concerns**	Before‐immediately	−19.78	−23.49 to −16.06	−2.11 (−2.59 to −1.61)	< 0.001
	Before—6 weeks later	−13.22	−17.81 to −8.62	−1.14 (−1.56 to 0.71)	< 0.001
	Immediately—6 weeks later	6.56	2.80 to 10.31	0.69 (0.28 to 1.09)	0.001
**Anxiety**	Before‐immediately	−10.84	−14.07 to −7.60	−1.32 (−1.76 to −0.89)	< 0.001
	Before—6 weeks later	−7.86	−10.87 to −4.84	−1.03 (−1.45 to −0.61)	< 0.001
	Immediately—6 weeks later	2.98	0.98 to 5.86	0.41 (0.01 to 0.80)	0.043

*Note*: Independent samples *t*‐test.

Due to the fact that the base value for the two previous information of puberty and mother's job variables was different in the two intervention and control groups, the ANCOVA test was performed to investigate the effect of these variables on the effectiveness of the intervention method. The results of the ANCOVA test showed that the body image concern score after the study was different between the two groups considering the baseline values and two variables puberty information and the mother's job and this difference was statistically significant (*p* < 0.001). Also, the ANCOVA test results indicated a statistically significant difference in anxiety scores post‐study between the intervention and control groups, when accounting for baseline values and two different variables between the two groups before the study (*p* < 0.001). The observed power showed that the sample size considered at the beginning of the study was appropriate to achieve the desired results. The effect size obtained for body image concern and anxiety, which were 0.56 and 0.41, respectively, indicates the greater effect of the intervention on the body image concern variable than anxiety (Table 5).

**TABLE 5:** Comparison between two groups for body image concern score and anxiety score after the intervention by adjusting to the baseline values, puberty information and mother's job among adolescent female students of Babol City.

Variable	*F*, df, *p* value^a^ (Effect of intervention)	*p* value (Basic value)	Adjusted *R* squared	Effect size	Observed power
**Body image concerns**	122.19, 1, < 0.001	< 0.001	0.63	0.56	1.00
**Anxiety**	65.32, 1, < 0.001	< 0.001	0.54	0.41	1.00

^a^ Based on ANCOVA model (the included variables were: basic value of dependent variable and group type).

## 4 Discussion

In this research, the effectiveness of pubertal health group counseling based on problem‐solving on body image concerns in adolescent girls of Babol City was designed and implemented. Also, students' anxiety was also investigated as a secondary outcome. The results of the present study showed that the level of body image concern and anxiety after the study significantly decreased in the intervention group compared to the control group. The reduction of anxiety and concern about body image in the intervention group was clinically significant.

The present study considered the group problem‐solving method for counseling adolescence girls, and teaching them this skill reduced their anxiety and worry. One of the methods that play a role in reducing puberty concerns is problem‐solving skills which is one of the vital skills for living in the present era (Koumleh et al. 2017). Problem‐solving is considered a purposeful and conscious activity (Sardarari and Ghorbanzadeh 2020). Considering cultural issues and taboos in society, along with the lack of addressing all adolescent problems and educating about them (Panjalipour et al. 2020), developing problem‐solving skills can help adolescents tackle challenges related to education. They have not been equipped with this problem‐solving approach. The impact of problem‐solving methods in addressing various issues is evident in the following studies.

In this regard, Hoseini et al. (2021) conducted a study in Tehran. Their findings showed the effectiveness of group counseling based on problem‐solving on girls' pubertal decreased health concerns, which was consistent with the present study (Hoseini et al. 2021). This convergence of findings strengthens the evidence base for utilizing problem‐solving interventions to address health‐related challenges during puberty. While Hoseini et al. (2021) focused on general pubertal health concerns, the present study extends this evidence by specifically examining the impact on body image concerns and anxiety, two critical aspects of adolescent well‐being. Other studies have also shown the positive effect of counseling based on problem‐solving faced with critical and difficult issues, including accepting the role of mother in mothers with premature babies (Rajabi et al. 2021), performance and sexual satisfaction in women after mastectomy (Bokaie et al. 2022), improved performance of adolescents with psychosis (Rapado‐Castro et al. 2019), mental health (Parikh et al. 2019), and life satisfaction of adolescents (Gál et al. 2022).

In contrast to the findings of Kheirollahi et al. (2017), who found no significant effect of a health belief model‐based intervention on pubertal health knowledge, the present study demonstrated a significant reduction in body image concerns and anxiety following problem‐solving group counseling. This discrepancy may be attributed to the differing theoretical frameworks and intervention strategies employed. The health belief model focuses on modifying health beliefs to influence behavior, while the problem‐solving approach equips adolescents with practical skills to actively manage their concerns (Bokaie et al. 2022). Similarly, while Nazarpour et al. (2020) found skill‐based health education to be more effective than traditional lectures in improving knowledge and attitudes about pubertal health, they observed no significant difference in performance between the groups after 2 months. This suggests that knowledge and attitude change may not always translate into behavioral change, and that the problem‐solving approach, with its emphasis on present‐focused action and adaptive strategy selection, may be more effective in promoting tangible improvements in well‐being.

The intervention carried out in the present study was able to reduce one of the problems that adolescents face during puberty, body image concerns. Body image concern is a problem that many people of different ages with different social and cultural differences are facing (Salarian Kaleji et al. 2022; Khoshyari Morad and Nik Amal 2021). This finding underscores the vulnerability of adolescent girls to negative body image perceptions during this period of significant physical and psychological change (Mercader‐Yus et al. 2018) and highlights the potential of problem‐solving interventions to mitigate these concerns by equipping them with the skills to evaluate and address their challenges effectively.

Adolescence is a critical period for the onset of psychiatric conditions like depression and anxiety (Boivin et al. 2017; Leen‐Feldner et al. 2008). The current study's finding that group counseling using problem‐solving techniques significantly reduced anxiety levels in adolescent girls aligns with a growing body of evidence supporting the efficacy of this approach for addressing anxiety in various adolescent populations (Ahvan and Mirzaei 2020; Michelson et al. 2022; Nawabi et al. 2020; H. Kazemi et al. 2016; Siraj Kermani and Akbari Amarghan 2016; Selsenik and Bibir 2018). This consistency across studies strengthens the rationale for implementing problem‐solving interventions to promote mental well‐being during this vulnerable developmental stage.

However, Qahraman (2016) reported that while teaching stress management skills and problem‐solving methods improved the mental health of adolescents, it did not significantly reduce their anxiety (Ghahraman and Zulfiqari Nia 2016). This seemingly contradictory finding may be attributable to the specific context of the intervention, which focused on exam anxiety. Exam anxiety is a multifaceted issue influenced by various factors, such as student interest, teacher–student relationships, and perceived importance of the subject matter. These factors may moderate the effectiveness of problem‐solving interventions in this specific domain. In contrast, the present study addressed general anxiety and body image concerns, which may be more amenable to the problem‐solving approach.

The present study had some limitations, including the lack of cooperation of parents and students in completing the questionnaires, and poor collaboration of the school staff in holding meetings with parents. Another limitation was the impossibility of random allocation technique.

The current study assessed sample size and confounding factors to minimize their impact. Evaluating intervention results in line with core values enhances the study's credibility. In addition, the significant reduction in body image concerns and anxiety through problem‐solving group counseling is clinically relevant. A further strength of this study is its focus on training future members of society. However, since the sample was drawn from two ecoculturally and economically distinct urban areas, and the sample size was deemed adequate through statistical analysis, the results demonstrate high generalizability.

To improve external validity, it is recommended to use a more diverse sample from various geographical locations and to include adolescent boys. In addition, conducting a long‐term follow‐up after the intervention would provide valuable insights into the lasting effects of the pubertal health group counseling intervention.

## 5 Conclusion

This research indicates that problem‐solving group counseling can effectively reduce body image concerns and anxiety among adolescent girls. Therefore, to promote adolescent health, it is recommended to implement problem‐solving interventions to educate this age group about puberty and its changes.

## Author Contributions

**Sana Nazmi**: conceptualization, methodology, writing – original draft, writing – review and editing, investigation, project administration, validation. **Hossein‐Ali Nikbakht**: methodology, software, writing – original draft, writing – review and editing, formal analysis, data curation. **Fereshteh Behmanesh**: conceptualization, methodology, supervision, writing – original draft, writing – review and editing, investigation, project administration, visualization, data curation. **Zeinab Gholamnia Shirvani**: methodology, writing – review and editing. **Alireza Azizi**: methodology, writing – review and editing.

## Ethics Statement

The study was approved by the Research Ethical Committee of Babol University of Medical Sciences (IR.MUBABOL.HRI.REC.1401.222). Written informed consent was taken from all the participants. All methods were carried out in accordance with relevant guidelines and regulations.

## Consent

The authors have nothing to report.

## Conflicts of Interest

The authors declare no conflicts of interest.

## Peer Review

The peer review history for this article is available at https://publons.com/publon/10.1002/brb3.70639

## Data Availability

The datasets used and/or analyzed during the current study are available from the corresponding author on reasonable request.

## References

[brb370639-bib-0001] Ahvan, L. , and P. Mirzaei . 2020. “The Effectiveness of Group Cognitive‐Social Problem Solving on Self‐Control and Anxiety in Girl Preschool Children.” Social Cognition 9, no. 2: 27–40.

[brb370639-bib-0002] Alsadat Fatemi, V. , A. Shafie Abadi , J. Khalatbari , and A. Farhangi . 2022. “The Effectiveness of Acceptance and Commitment Therapy on Communication Skills of Female Students of High School.” Journal of Applied Family Therapy 3, no. 4: 169–184.

[brb370639-bib-0003] Ataie Moghanloo, V. , M. M. BassakNezhad S , and R. Ataie Moghanloo . 2014. “The Effect of Adolescent Mental Health Education on Aggression and Fear of Body Image in the Second Grade Male Secondary School Students in Ahvaz City.” *Health and Health Magazine*.

[brb370639-bib-0004] Baharvand, P. , F. Malekshahi , and P. Mahdiyan . 2020. “Prevalence of Body Image Concern Among School Girls Aged 12–17 Years in Iran.” Journal of Education and Health Promotion 9, no. 1: 210. 33062743 10.4103/jehp.jehp_259_20 PMC7530431

[brb370639-bib-0005] Barkhordari‐Sharifabad, M. , S. Vaziri‐Yazdi , and M. Barkhordari‐Sharifabad . 2020. “The Effect of Teaching Puberty Health Concepts on the Basis of a Health Belief Model for Improving Perceived Body Image of Female Adolescents: A Quasi‐Experimental Study.” BMC Public Health 20, no. 1: 1–7. 32197594 10.1186/s12889-020-08482-2 PMC7083033

[brb370639-bib-0006] Basak Nejad, S. , and M. Ghaffari . 2007. “The Relationship Between Fear of Physical Deformity and Psychological Disorders in Students.” Behavioral Sciences 2, no. 1: 179–188.

[brb370639-bib-0007] Bassaknezhad, S. , and V. Ataie Moghanloo . 2013. “The Effectiveness of Puberty Mental Health Training on Fear of Body Image and Adjustment in High School Male Students.” Journal of Research and Health 3, no. 1: 269–277.

[brb370639-bib-0008] Bibiloni, M. D. M. , J. L. Coll , J. Pich , A. Pons , and J. A. Tur . 2017. “Body Image Satisfaction and Weight Concerns Among a Mediterranean Adult Population.” BMC Public Health 17: 1–11. 28061761 10.1186/s12889-016-3919-7 PMC5217589

[brb370639-bib-0009] Boivin, J. R. , D. J. Piekarski , J. K. Wahlberg , and L. Wilbrecht . 2017. “Age, Sex, and Gonadal Hormones Differently Influence Anxiety‐and Depression‐Related Behavior During Puberty in Mice.” Psychoneuroendocrinology 85: 78–87. 28837909 10.1016/j.psyneuen.2017.08.009 PMC6596309

[brb370639-bib-0010] Bokaie, M. , O. Firouzabadi , and A. Joulaee . 2022. “The Effectiveness of Group Problem‐Solving Therapy on Women's Sexual Function and Satisfaction After Mastectomy Surgery.” BMC Women's Health 22, no. 1: 1–7. 35197028 10.1186/s12905-022-01628-x PMC8867677

[brb370639-bib-0011] Crerand, C. E. , D. B. Sarwer , A. E. Kazak , A. Clarke , and N. Rumsey . 2017. “Body Image and Quality of Life in Adolescents With Craniofacial Conditions.” Cleft Palate‐Craniofacial Journal 54, no. 1: 2–12. 10.1597/15-167 PMC5603909 26751907

[brb370639-bib-0012] Darabi, F. , M. H. Kaveh , F. K. Farahani , M. Yaseri , F. Majlessi , and D. Shojaeizadeh . 2017. “The Effect of a Theory of Planned Behavior‐Based Educational Intervention on Sexual and Reproductive Health in Iranian Adolescent Girls: A Randomized Controlled Trial.” Journal of Research in Health Sciences 17, no. 4: 400.29233954

[brb370639-bib-0013] Djalalinia, S. , F. R. Tehrani , H. M. Afzali , F. Hejazi , and N. Peykari . 2012. “Parents or School Health Trainers, Which of Them Is Appropriate for Menstrual Health Education?” International Journal of Preventive Medicine 3, no. 9: 622. 23024851 PMC3445278

[brb370639-bib-0014] Eslamimehr, F. , F. Rakhshani , A. Ramezan Khani , and S. Khodakarim . 2017. “The Examination of the Effectiveness of an Educational Intervention Based on the Planned Behavior Theory on Improving Pubertal Health Behavior in Female High School Students.” International Journal of Pediatrics 5, no. 9: 5643–5654.

[brb370639-bib-0015] Gál, Z. , L. Kasik , S. Jámbori , J. B. Fejes , and K. Nagy . 2022. “Social Problem‐Solving, Life Satisfaction and Well‐Being Among High School and University Students.” International Journal of School & Educational Psychology 10, no. 1: 170–180.

[brb370639-bib-0016] George, N. , A. R. Johnson , A. Lobo , C. Simily , S. Pousiya , and T. Agrawal . 2018. “Health Problems and Health Seeking Behavior Among School‐Going Adolescents in a Rural Area in South Karnataka.” Journal of Indian Association for Child and Adolescent Mental Health 14: 50–65.

[brb370639-bib-0017] Ghahraman, M. , and M. Zulfiqari Nia . 2016. “The Effect of Teaching Stress Management Skills and Problem Solving Methods on Mental Health and Exam Anxiety.” Paper presented at the World Conference of Psychology and Educational Sciences, Law and Social Sciences at the Beginning of the Third Millennium, Shiraz, November 10.

[brb370639-bib-0018] Golchin, N. A. H. , Z. Hamzehgardeshi , M. Fakhri , and L. Hamzehgardeshi . 2012. “The Experience of Puberty in Iranian Adolescent Girls: A Qualitative Content Analysis.” BMC Public Health 12, no. 1: 698. 22925369 10.1186/1471-2458-12-698 PMC3488498

[brb370639-bib-0019] Haque, S. E. , M. Rahman , K. Itsuko , M. Mutahara , and K. Sakisaka . 2014. “The Effect of a School‐Based Educational Intervention on Menstrual Health: An Intervention Study Among Adolescent Girls in Bangladesh.” BMJ Open 4, no. 7: e004607. 10.1136/bmjopen-2013-004607 PMC4091465 24993753

[brb370639-bib-0020] Hoseini, Z. , N. Akbari Torkestani , A. Majidi , and A. Moslemi . 2021. “The Effects of Problem‐Solving‐Based Puberty Group Counseling on Adolescent Girls' Health Concerns.” Journal of Arak University of Medical Sciences 24, no. 4: 470–481.

[brb370639-bib-0022] Junne, F. , S. Zipfel , B. Wild , et al. 2016. “The Relationship of Body Image With Symptoms of Depression and Anxiety in Patients With Anorexia Nervosa During Outpatient Psychotherapy: Results of the ANTOP Study.” Psychotherapy 53, no. 2: 141–151. 27267500 10.1037/pst0000064

[brb370639-bib-0023] Kay, R. , T. MacDonald , and M. DiGiuseppe . 2019. “A Comparison of Lecture‐Based, Active, and Flipped Classroom Teaching Approaches in Higher Education.” Journal of Computing in Higher Education 31: 449–471.

[brb370639-bib-0024] Kazemi, Z. , D. Shojaeezadeh , and Z. Jalili . 2020. “The Effect of Educational Interventions Based on Health Belief Model (HBM) on Puberty Health Behaviors in Tehran's Female Elementary Students, 2019.” Iranian Journal of Health Education and Health Promotion 8, no. 2: 142–159.

[brb370639-bib-0025] Kazemi, H. , M. Waziri , and A. Abedi . 2016. “The Effectiveness of Problem Solving Training on Exam Anxiety and Social Anxiety of Primary School Students.” Social Cognition 5, no. 1: 100–112.

[brb370639-bib-0026] Keshavarze, M 2020. “Maturity and Identity Crisis in Adolescence and Its Role in Making Friends.” Psychology and Behavioral Sciences of Iran 33, no. 5: 132–142.

[brb370639-bib-0027] Khazaie, S. , and M. Riahi . 2021. “A Qualitative Study of Adolescent Body Identity (High School Girls in Boroujerd).” Journal of Applied Sociology 32, no. 1: 107–134.

[brb370639-bib-0028] Kheirollahi, F. , Z. Rahimi , S. Arsang‐Jang , G. Sharifirad , P. Sarraf , and Z. Gharlipour . 2017. “Puberty Health Status Among Adolescent Girls: A Model‐Based Educational Program.” International Journal of Pediatrics 5, no. 7: 5369–5378.

[brb370639-bib-0029] Kheyrkhah, M. , H. Mokarie , L. Neisani , and F. Hoseini . 2013. “The Impact of Puberty Health Education on Self Concept of Adolescents.” Iranian Journal of Nursing Research 8, no. 3: 47–57.

[brb370639-bib-0030] Khoshyari Morad, S. , and M. Nik Amal . 2021. “The Effectiveness of Anxiety Relief for Teenage Girls Facing Puberty in Orphanages.” [In Persian.] Siklus: Journal Research Midwifery Politeknik Tegal 10, no. 1: 53–56.

[brb370639-bib-0031] Koumleh, S. E. , E. Naderi , and M. S. Naraghi . 2017. “The Role of Problem Solving Skills in Emergence of Healthy and Positive Behaviors: Development and Evaluation of an Optimal Model for Social Studies for Primary School Curriculum.” Payesh (Health Monitor) 16, no. 1: 39–51.

[brb370639-bib-0032] Kristjánsdóttir, H. , P. Sigurðardóttir , S. Jónsdóttir , G. Þorsteinsdóttir , and J. Saavedra . 2019. “Body Image Concern and Eating Disorder Symptoms Among Elite Icelandic Athletes.” International Journal of Environmental Research and Public Health 16, no. 15: 2728. 31370175 10.3390/ijerph16152728 PMC6696614

[brb370639-bib-0033] Leen‐Feldner, E. W. , L. E. Reardon , C. Hayward , and R. C. Smith . 2008. “The Relation Between Puberty and Adolescent Anxiety: Theory and Evidence.” In Anxiety in Health Behaviors and Physical Illness, 155–179. Springer Nature.

[brb370639-bib-0034] Lenhard, W. , and A. Lenhard . 2022. Computation of Effect Sizes. Psychometrica.

[brb370639-bib-0035] Littleton, H. L. , D. Axsom , and C. L. Pury . 2005. “Development of the Body Image Concern Inventory.” Behaviour Research and Therapy 43, no. 2: 229–241. 15629752 10.1016/j.brat.2003.12.006

[brb370639-bib-0068] Mastorci, F. , M. F. L. Lazzeri , C. Vassalle , and A. Pingitore . 2024. “The Transition from Childhood to Adolescence: Between Health and Vulnerability.” Children (Basel, Switzerland) 11, no. 8: 989. 39201923 10.3390/children11080989 PMC11352511

[brb370639-bib-0036] Mazloomy Mahmoodabad, S. , S. Norouzi , A. Norouzi , A. Hajizadeh , and A. ZareA . 2013. “Effect of Health Belief Model in Adopting Prevention and Control of Health Behaviors During Puberty High School Students in Ardakan City.” Tolooebehdasht 12, no. 1: 56–66.

[brb370639-bib-0037] Mercader‐Yus, E. , M. C. Neipp‐López , P. Gómez‐Méndez , et al. 2018. “Anxiety, Self‐Esteem and Body Image in Girls With Precocious Puberty.” Revista Colombiana De Psiquiatría (English Ed) 47, no. 4: 229–236. 10.1016/j.rcp.2017.05.013 30286845

[brb370639-bib-0038] Michelson, D. , E. Hodgson , A. Bernstein , B. F. Chorpita , and V. Patel . 2022. “Problem Solving as an Active Ingredient in Indicated Prevention and Treatment of Youth Depression and Anxiety: An Integrative Review.” Journal of Adolescent Health 71: 390–405. 10.1016/j.jadohealth.2022.05.005 35803863

[brb370639-bib-0039] Michelson, D. , K. Malik , R. Parikh , et al. 2020. “Effectiveness of a Brief Lay Counsellor‐Delivered, Problem‐Solving Intervention for Adolescent Mental Health Problems in Urban, Low‐Income Schools in India: A Randomised Controlled Trial.” Lancet Child & Adolescent Health 4, no. 8: 571–582. 32585185 10.1016/S2352-4642(20)30173-5 PMC7386943

[brb370639-bib-0040] Mohammadi, M. , A. Ziapour , M. Mahboubi , et al. 2014. “Performance Evaluation of Hospitals Under Supervision of Kermanshah Medical Sciences Using Pabonlasoty Diagram of a Five‐Year Period (2008–2012).” Life Science Journal 11: 77–81.

[brb370639-bib-0041] Mokari, H. , S. Khaleghparast , and L. N. Samani . 2016. “Impact of Puberty Health Education on Anxiety of Adolescents.” International Journal of Medical Research & Health Sciences 5, no. 5: 284–291.

[brb370639-bib-0042] Nawabi, M. , R. Guderzi , and D. Kurdistan . 2020. “Comparing the Effectiveness of Happiness Training and Problem Solving Skills Training on Social Anxiety and Self‐Compassion of Adolescents.” Islamic Lifestyle With a Focus on Health 4, no. 4: 103–110.

[brb370639-bib-0043] Nazarpour, S. , Z. Arabi , M. Simbar , Z. Keshavarz , and A. R. Baghestani . 2020. “A Comparison Between the Skills‐Based Education With a Lecture‐Based Education on Female Adolescents' knowledge, Attitude and Practice About Health in Puberty: A Randomized Trail Study.” Advances in Nursing & Midwifery 29: 15–23.

[brb370639-bib-0044] Nazmi, S. , H.‐A. Nikbakht , Z. Gholamnia‐Shirvani , F. Behmanesh , and A. Azizi . 2024. “The Effectiveness of Pubertal Health Group Counseling Based on Problem‐Solving, on Body Image Concerns and Anxiety in Adolescent Girls: Study Protocol.” Journal of Education and Health Promotion 13, no. 1: 215. 39297101 10.4103/jehp.jehp_738_23 PMC11410170

[brb370639-bib-0045] Nouri, M 2010. “The Impact of Peer‐Based Educational Approaches on Girls' Physical Practice of Pubertal Health.” Journal of Arak University of Medical Sciences 12, no. 4: 129–135.

[brb370639-bib-0046] Nozari, R. A. , A. Farshbaf‐Khalili , N. Sattarzadeh , and M. A. Jafarabadi . 2019. “The Effect of Counseling on Menstrual Hygiene, Physical Activity, and Nutritional Status of Female Adolescent Students: A Randomized Controlled Field Trial.” Crescent Journal of Medical & Biological Sciences 6, no. 3: 393–402.

[brb370639-bib-0047] Nulty, D. D 2008. “The Adequacy of Response Rates to Online and Paper Surveys: What Can be Done? Assessment & Evaluation in Higher Education.” Assessment & Evaluation in Higher Education 33, no. 3: 301–314.

[brb370639-bib-0048] Olatunji, B. O. , B. J. Deacon , J. S. Abramowitz , and D. F. Tolin . 2006. “Dimensionality of Somatic Complaints: Factor Structure and Psychometric Properties of the Self‐Rating Anxiety Scale.” Journal of Anxiety Disorders 20, no. 5: 543–561. 16198532 10.1016/j.janxdis.2005.08.002

[brb370639-bib-0049] Panjalipour, S. , Z. Bostani Khalesi , S. Rezaei Chamani , and E. Kazemnejad Leili . 2020. “Prioritizing the Healthcare Needs of Adolescent Girls in Iran.” Journal of Guilan University of Medical Sciences 29, no. 3: 58–71.

[brb370639-bib-0050] Parikh, R. , D. Michelson , K. Malik , et al. 2019. “The Effectiveness of a Low‐Intensity Problem‐Solving Intervention for Common Adolescent Mental Health Problems in New Delhi, India: Protocol for a School‐Based, Individually Randomized Controlled Trial With an Embedded Stepped‐Wedge, Cluster Randomized Controlled Recruitment Trial.” Trials 20, no. 1: 1–18. 31533783 10.1186/s13063-019-3573-3 PMC6751586

[brb370639-bib-0051] Raghibi, M. , H. Sheikh , Y. Shamsollahzadeh , and M. Jalmbadani . 2019. “Investigating the Mediating Role of Identity Styles on Body Image Concerns and Related Factors in Adolescents Referred to Zahedan Health Centers.” Journal of Health Research in Community 5, no. 3: 83–93.

[brb370639-bib-0052] Rahbari, N. , B. Jalil , and S. M. Hasan . 2017. “The Effectiveness of Teaching Problem‐Solving and Daring Skills on Changing the Attitude of Adolescent Girls Towards Substance Abuse.” Research Addiction 12: 11–26.

[brb370639-bib-0053] Rajabi, A. , A. Maleki , M. Dadashi , and F. Karami Tanha . 2021. “The Effect of Problem‐Solving‐Approach‐Based Counselling on Maternal Role Adaptation in Women With Late Preterm Infant: A Randomized Controlled Trial.” Journal of Caring Sciences 10, no. 2: 62–69. 34222114 10.34172/jcs.2021.013 PMC8242297

[brb370639-bib-0054] Rapado‐Castro, M. , C. Moreno , A. Ruíz‐Sancho , F. Camino , C. Arango , and M. Mayoral . 2019. “Role of Executive Function in Response to a Problem Solving Based Psychoeducational Intervention in Adolescents With Psychosis: The PIENSA Trial Revisited.” Journal of Clinical Medicine 8, no. 12: 2108. 31810220 10.3390/jcm8122108 PMC6947315

[brb370639-bib-0055] Reardon, L. E. , E. W. Leen‐Feldner , and C. Hayward . 2009. “A Critical Review of the Empirical Literature on the Relation Between Anxiety and Puberty.” Clinical Psychology Review 29, no. 1: 1–23. 19019513 10.1016/j.cpr.2008.09.005 PMC2652567

[brb370639-bib-0056] Sahebi, A. , and S. R. Salari . 2005. “Validation of Depression Anxiety Stress Scale (DASS‐21) for Iranian Population.” Journal of Iranian Psychologists 1, no. 4: 36–54.

[brb370639-bib-0057] Salarian Kaleji, Z. , H. Poursharifi , B. Dolatshahi , and F. Momeni . 2022. “The Relationship Between Body Image Victimization Experiences and Binge Eating Symptoms: The Mediating Role of Body Image Shame and Self‐Criticism.” Iranian Journal of Psychiatry and Clinical Psychology 28, no. 1: 48–61.

[brb370639-bib-0058] Santamaria, S 2020. “ADAMTS‐5: A Difficult Teenager Turning 20.” International Journal of Experimental Pathology 101, no. 1–2: 4–20. 32219922 10.1111/iep.12344 PMC7306899

[brb370639-bib-0059] Sardarari, B. , and P. Ghorbanzadeh . 2020. “The Effectiveness of Teaching Problem Solving Skills on Positive and Negative Academic Emotions of Sixth Grade Students.” Research in Educational Sciences and Counseling 1398, no. 11: 125–144.

[brb370639-bib-0060] Selsenik, B. , and R. Bibir . 2018. “Problem Solving Training for Home‐Dependent Adolescents With Anxiety Disorders.” Social and Behavioral Sciences 2014: 101.

[brb370639-bib-0061] Sharifi, S. M. , A. Omidi , and B. Marzban . 2016. “The Impact of Instagram Use on Body Image Concerns Among Iranian University Female Students: A Phenomenological Approach.” International Journal of Academic Research in Psychology 3: 26–36.

[brb370639-bib-0062] Shoraka, H. , A. Amirkafi , and B. Garrusi . 2019. “Review of Body Image and Some of Contributing Factors in Iranian Population.” International Journal of Preventive Medicine 10, no. 1: 19. 30820306 10.4103/ijpvm.IJPVM_293_18 PMC6390429

[brb370639-bib-0063] Siraj Kermani, H. , and H. Akbari Amarghan . 2016. “The Effect of Problem Solving Skill Training on Exam Anxiety of Male Students of the Second Period Public High Schools in the 7th District of Mashhad.” Paper presented at The Fourth National Conference on Counseling And Mental Health.

[brb370639-bib-0064] WHO . 2021. “Mental Health of Adolescents.” https://www.who.int/news‐room/fact‐sheets/detail/adolescent‐mental‐health.

[brb370639-bib-0065] Ziapour, A. , M. Sharma , N. NeJhaddadgar , A. Mardi , and S. S. Tavafian . 2020. “Educational Needs Assessment Among 10–14‐year‐old Girls About Puberty Adolescent Health of Ardebil.” Archives of Public Health 78: 1–6. 32025298 10.1186/s13690-019-0388-3 PMC6996169

[brb370639-bib-0066] Ziapour, A. , M. Sharma , N. NeJhaddadgar , A. Mardi , and S. S. Tavafian . 2021. “Study of Adolescents' Puberty, Adolescence Training Program: The Application of Intervention Mapping Approach.” International Quarterly of Community Health Education 42, no. 1: 5–14. 32903158 10.1177/0272684X20956485

[brb370639-bib-0067] Zung, W. W 1971. “A Rating Instrument for Anxiety Disorders.” Psychosomatics: Journal of Consultation and Liaison Psychiatry 12: 371–379. 10.1016/S0033-3182(71)71479-0 5172928

